# Evidence for *Escherichia coli* DcuD carrier dependent F_O_F_1_-ATPase activity during fermentation of glycerol

**DOI:** 10.1038/s41598-019-41044-0

**Published:** 2019-03-12

**Authors:** L. Karapetyan, A. Valle, J. Bolivar, A. Trchounian, K. Trchounian

**Affiliations:** 1 Department of Biochemistry, Microbiology and Biotechnology, Faculty of Biology, Yerevan State University, 1 A. Manoogian str., 0025 Yerevan, Armenia; 2 Scientific-Research Institute of Biology, Yerevan State University, 1 A. Manoogian str., 0025 Yerevan, Armenia; 3 Microbial Biotechnologies and Biofuel Innovation Center, Yerevan State University, 1 A. Manoogian str., 0025 Yerevan, Armenia; 4 Department of Biomedicine, Biotechnology and Public Health-Biochemistry and Molecular Biology, Institute of Biomolecules (INBIO), University of Cádiz, Avda. República Saharui s/n, 11510 Puerto Real, Cádiz Spain

## Abstract

During fermentation *Escherichia coli* excrete succinate mainly via Dcu family carriers. Current work reveals the total and *N,N’*-dicyclohexylcarbodiimide (DCCD) inhibited ATPase activity at pH 7.5 and 5.5 in *E. coli* wild type and *dcu* mutants upon glycerol fermentation. The overall ATPase activity was highest at pH 7.5 in *dcuABCD* mutant. In wild type cells 50% of the activity came from the F_O_F_1_-ATPase but in *dcuD* mutant it reached ~80%. K^+^ (100 mM) stimulate total but not DCCD inhibited ATPase activity 40% and 20% in wild type and *dcuD* mutant, respectively. 90% of overall ATPase activity was inhibited by DCCD at pH 5.5 only in *dcuABC* mutant. At pH 7.5 the H^+^ fluxes in *E. coli* wild type, *dcuD* and *dcuABCD* mutants was similar but in *dcuABC* triple mutant the H^+^ flux decreased 1.4 fold reaching 1.15 mM/min when glycerol was supplemented. In succinate assays the H^+^ flux was higher in the strains where DcuD is absent. No significant differences were determined in wild type and mutants specific growth rate except *dcuD* strain. Taken together it is suggested that during glycerol fermentation DcuD has impact on H^+^ fluxes, F_O_F_1_-ATPase activity and depends on potassium ions.

## Introduction

*Escherichia coli* transport and use diverse C_4_-dicarboxylates (succinate, malate, aspartate or fumarate) in antiport manner or symport with H^+^ during aerobic or anaerobic growth. Among known C_4_-dicarboxylate transporters are DctA as well as the Dcu family DcuA, DcuB, DcuC and the putative DcuD transporter^1^. It is well established that DctA is important for aerobic growth on C_4_-dicarboxylates. Dcu carriers are different from DctA and form a separate group. It has been suggested that DcuA, encoded by *dcuA* gene, catalyzes the uptake of succinate or fumarate and is active either in aerobic or anaerobic conditions. The other carriers (DcuB, DcuC) are expressed only under anaerobic conditions^1,2^. It was clearly shown that DcuB is the major C_4_-dicarboxylate carrier under anoxic conditions. DcuC, encoded by the *dcuC* gene, is synthesized under anaerobic conditions and during glucose fermentative conditions is suggested to function preferably as an efflux carrier^1,3^. Gene expression data showed that fumarate or other C_4_-dicarboxylates might increase the gene expression level of several carriers^4^. But substitution of glucose by glycerol did not affect *dcuC* expression, thus it can be assumed that *dcuC* is not subject to catabolite repression and DcuC is needed for succinate efflux during glucose fermentation^1,3^. To be critical, it must be mentioned that glycerol substituted to glucose was used in the medium with the presence of fumarate, and glucose fermentation cannot be compared to glycerol fermentation, as fumarate respiration takes place. Moreover, these carrier proteins are dependent on external pH and lack of Dcu function in the cells resulted in aerobic growth on succinate when external pH was below 6.0^1^.

The fourth DcuD carrier, encoded by *dcuD* gene (formerly *yhcL*), is not expressed under most of the conditions tested and its physiological role is still unknown^5^. Lately, a work demonstrated that in *dcuD* mutant the product yields of molecular hydrogen H_2_ and ethanol are improved^6^. Moreover, by deletion *dcuB* and *dcuC* but not *dcuA* and *dcuD* genes resulted in the increase of succinate production by 34%^3^. In addition, during glucose fermentation the deletions of *dcuB* and *dcuC* resulted in 90% decrease of succinate titer suggesting that DcuB and DcuC are responsible for succinate efflux under the latest conditions^3^.

A decade ago it was shown that glycerol can be fermented by *E. coli* under anaerobic conditions at different pH values^7–9^. Depending on external pH fermentation end products are various, and key bioenergetics parameters such as membrane potential, pH gradient and thus proton motive force (Δµ_H_^+^) values are also different, compared to glucose fermentative conditions^10–13^. One of the key enzymes for *E. coli* growth under anaerobic conditions is the proton translocating F_O_F_1_-ATPase, which is the main Δµ_H_^+^ generator. It has been experimentally shown that the F_O_F_1_-ATPase activity is necessary for the activity of membrane bound [Ni-Fe] hydrogenase (Hyd) enzymes, which are responsible for H_2_ metabolism and potassium (K^+^) transport enzymes such as Trk or others^13,14^. F_O_ subunit of proton F_O_F_1_-ATPase is located inside the cytoplasmic membrane and contains a, b, and c subunits^15,16^. The extra-membranous F_1_ subunit is attached to the F_O_ part, and in F_1_ ATP hydrolysis takes place under fermentative conditions^15^. Particularly, during glucose or glycerol fermentation Hyd-1 or Hyd-2 depend on the active F_O_F_1_-ATPase. Moreover, this link or metabolic cross-talk depends on external pH and other conditions^17^. The results were obtained by inhibiting the proton F_O_F_1_-ATPase with *N,N’*-dicyclohexylcarbodiimide (DCCD), a specific inhibitor of the *E. coli* F_O_F_1_-ATPase under anaerobic conditions^18^, or applying *atp* (DK8) mutant which do not have F_O_F_1_-ATPase^19^.

During glycerol fermentative conditions, the role of different carriers such as Dcu is not known because when the experiments were carried out with glycerol and fumarate^1–3^ the metabolism goes to fumarate respiration but not to glycerol fermentation. At that time glycerol fermentation was not known yet. So the current work describes novel properties of Dcu carriers and, especially previously unknown role of DcuD during glycerol fermentation at pH 7.5 and 5.5.

## Results and Discussion

### ATPase activity and H^+^ fluxes of E. coli wild type and dcu mutants at pH 7.5 and pH 5.5

In 2006 Gonzalez group^7^ experimentally demonstrated that *E. coli* can ferment glycerol at slightly acidic and further by our group at slightly alkaline pHs^9^. Moreover, responsible Hyd enzymes and relationship between these enzymes with main enzyme of bioenergetic relevance – the proton F_O_F_1_-ATPase during fermentation have been determined^11^. To understand what is the role of C_4_-dicarboxylate carriers (Dcu) during glycerol fermentation the activity of proton F_O_F_1_-ATPase has been investigated. For this objective, total and DCCD inhibited ATPase activity at pH 7.5 and pH 5.5 has been defined. It is well established that DCCD is the specific inhibitor of F_O_F_1_-ATPase under anoxic conditions^18^.

**Figure 1:**
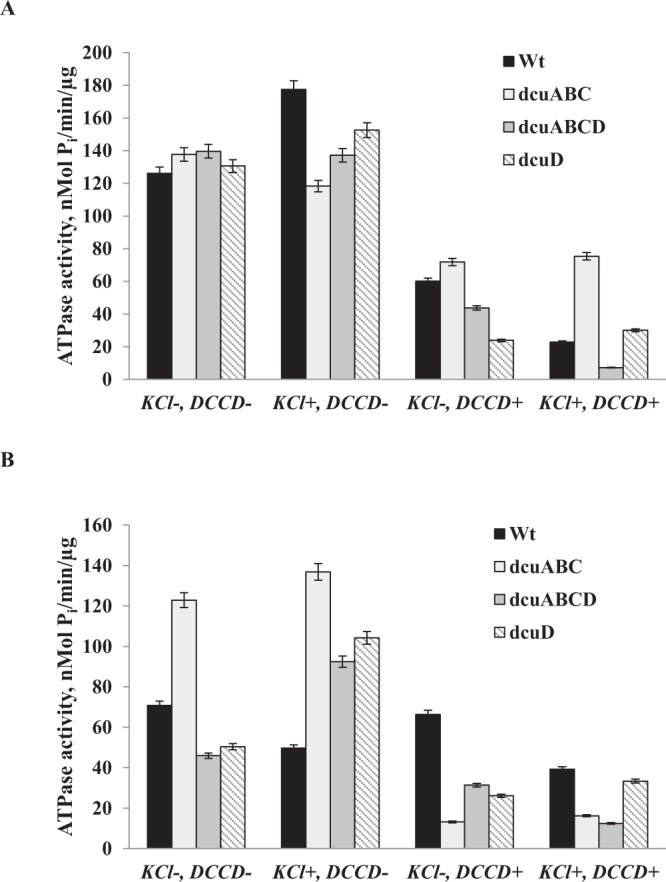
ATPase activity of membrane vesicles of *E. coli* BW25113 wild type, *dcuABC*, *dcuABCD*, *dcuD* mutant strains at pH 7.5 (**A**) and pH 5.5 (**B**). The DCCD (0.1 mM) was added into the assay medium when indicated. K^+^ (100 mM) was added in the assays when shown. The assays pH was the same as growth pH. Bacteria were grown at pH 7.5 or pH 5.5 in the presence of 10 g L^−1^ glycerol as carbon source at 37 °C. For the strains see Table 1; for others, see Methods.

The highest total ATPase activity was determined at pH 7.5 in *dcuABCD* mutant membrane vesicles resulting in 139.6 nMol P_i_/(min µg protein) (Fig. 1A) compared to wild type and *dcu* mutants. At pH 7.5 in wild type cells the total ATPase activity was higher by 44% compared to the cells grown at acidic pH 5.5 (compare Fig. 1A,B). In order to indicate the contribution of proton F_O_F_1_-ATPase in the total ATPase activity DCCD-inhibited ATPase activity was determined (see Methods). It was shown that at pH 7.5 in wild type and *dcuABC* mutant membrane vesicles DCCD the ATPase activity was inhibited 2 and 1.9 fold, respectively, suggesting that F_O_F_1_ contributed to total ATPase activity by 50% under the conditions mentioned. Interestingly, it was shown that in *dcuABCD* mutant DCCD inhibited by 22% more the ATPase activity compared to wild type and *dcuABC* triple mutant. But surprisingly in *dcuD* single mutant the DCCD inhibition was much higher and 82% of total ATPase activity came from F_O_F_1_ (see Fig. 1A).

The data suggest that there might be some relationship or interaction between DcuD protein and the F_O_F_1_-ATPase at pH 7.5. Particularly, it had been reviewed^1^ that during respiration with the transport of succinate^2^-3H^+^ are symported but under fermentative conditions the amount of H^+^ that are symported during succinate efflux is not known yet. The interrelationship between DcuD and F_O_F_1_ can take place if the DcuB and DcuC efflux succinate as well as the DcuD symport protons via interacting with F_O_F_1_. Similar interaction of *E. coli* potassium transport Trk system with F_O_F_1_ had been shown before^12–14,20^. In addition, it was shown that Na^+^ ions were important for the transport of C_4_ dicarboxylates in *W. succinogenes* and the absence of Na^+^ ions during fumarate respiration disturbs generation of proton motive force^21^. Moreover, it is possible that DcuD interact with F_O_F_1_ via thiol groups by having dithiol-disulfide interchange and by this way translocate protons. The possibility of the involvement of thiol groups in the above mentioned reaction was reviewed before^14^. In addition, recently “hydrogenase complex” idea as “proton sensor” had been proposed and in this model Dcu carriers might interact to regulate the proton gradient^22^.

**Figure 2:**
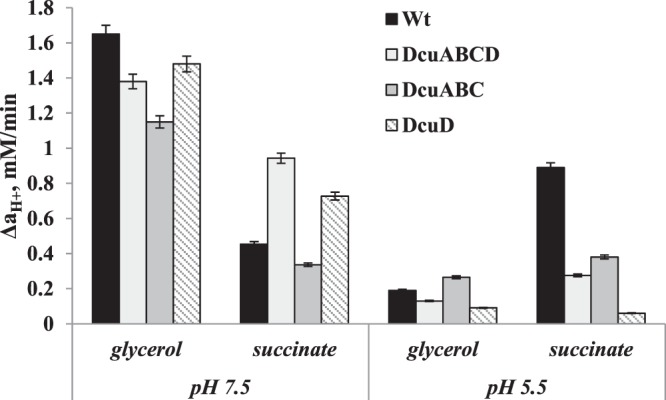
H^+^ efflux by whole cells of *E. coli* wild type and *dcu* mutants during glycerol fermentation at pH 7.5 and pH 5.5. In assays glycerol was used in the same concentration as in growth medium and succinate was added in concentration of 5 mM. For mutant strains, see Table 1; for the others, see the Methods section and the legend to Fig. 1.

The role of DcuD in proton translocation suggested was also confirmed by the H^+^ flux determination in this mutant (Fig. 2). It was clearly demonstrated that when cells were grown on glycerol, and in the assays glycerol was added no significant differences had been detected in the *dcuD* and *dcuABCD* mutants compared to wild type regarding the H^+^ efflux (see Fig. 2). Only in *dcuABC* mutant, where only dcuD is present, the H^+^ efflux was decreased by 26%. This suggests that DcuD might be involved in H^+^ efflux thus regulating the transmembrane pH gradient via interacting with other systems.

But when succinate was added in the assays the H^+^ fluxes in *dcuD* and *dcuABCD* mutants were higher by 1.6 and 2.07 folds, respectively, compared to wild type at pH 7.5. From the data obtained it is suggested that when DcuD is absent other Dcu carriers or membrane systems pump H^+^ out. There might be therefore some compensatory H^+^ pumping mechanism in Dcu carriers, which must be more deeply investigated to understand the likely compensatory function. Similar compensatory uptake or producing functions have been suggested for Hyd enzymes or for formate uptake or export Foc channels^23,24^. In addition, externally added succinate enters the cell in the absence of Dcu family carriers, which could indicate, in accordance with previously shown possibilities, that other C_4_ uptake systems can take the role of Dcu or other carriers^1,2,6^. But during anaerobic conditions there might be limited possibility of compensatory uptake functions of C_4_ uptake, an issue that must be further analyzed.

When cells were grown at pH 5.5 the highest total ATPase activity was obtained in *dcuABC* mutant membrane vesicles resulting in 122.84 nMol P_i_/(min µg protein) (Fig. 1B) compared to that obtained in wild type and the *dcu* mutants. In *dcuD* and *dcuABCD* mutants the total ATPase activity was decreased by 29% and 35%, respectively, compared to wild type (see Fig. 1B). DCCD inhibited ATPase activity mainly in *dcuABC* and *dcuD* mutants by 89% and 48%, respectively. No inhibition was determined in wild type and DCCD inhibited the ATPase activity by 31% in *dcuABCD* mutant. These results suggest that the mutations might affect the F_O_F_1_-ATPase conformational change or there must be a direct or indirect link with F_O_F_1_ at this pH also. Moreover, the H^+^ flux measured in whole cells showed that at pH 5.5 when glycerol was added the flux was very low in wild type and mutant cells. Addition of succinate in assays resulted in increase of H^+^ flux reaching 0.9 mM/min in wild type but not mutant cells compared to the assays supplemented with glycerol where the H^+^ flux was 0.19 mM/min (see Fig. 2). In all *dcu* mutants the H^+^ flux was lowered but only in *dcuD* single mutant it was absent which suggest that at low pH, DcuD mainly contribute to H^+^ efflux across the membrane during glycerol fermentation. As the role of DcuD is not clear at all, it might be possible that it is involved in inter-membrane proton translocation and depending on pH either it pumps H^+^ out of the cell or translocates it to other membrane bound enzymes or transport systems.

### Role of potassium ions in ATPase activity of E. coli wild type and dcu mutants membrane vesicles at pH 7.5 and pH 5.5

Earlier in many papers it has been described that K^+^ have significant role in F_O_F_1_-ATPase activity^20,25^. In addition, as stated above and model was proposed according to which K^+^ transport Trk system, Hyd-4 interacts with F_O_F_1_-ATPase and forms protein-protein complex at pH 7.5 during glucose fermentation^14^. Moreover, recently, it was shown that during mixed carbon (glucose, glycerol and formate) fermentative conditions formate dehydrogenase (Fdh) has some link or relationship with F_O_F_1_ depending on K^+^ at pH 5.5^26^. All this suggests that there must be some effect of K^+^ on C_4_-dicarboxylate carriers such as the anaerobic Dcu family.

In order to reveal the role of K^+^, cells membrane vesicles total and DCCD inhibited ATPase activity was determined in the presence of K^+^ (100 mM) (see Fig. 1). It was shown that K^+^ had stimulatory effect on wild type cells at pH 7.5 but not at pH 5.5. Especially, at pH 7.5 total ATPase activity was stimulated by 40% and DCCD inhibited more F_O_F_1_-ATPase in the presence of K^+^ which is in good conformity with stimulatory effect of K^+^ on F_O_F_1_ during glucose fermentation^20,25,27^. But when analyzing the mutants only in *dcuD* mutant, K^+^ stimulated the total ATPase activity by 17% but not DCCD inhibited one (see Fig. 1). DCCD totally inhibited ATPase activity only in *dcuABCD* mutant. The data clearly demonstrate that K^+^ affects C_4_-dicarboxylate Dcu carrier system and there is a link between F_O_F_1_, Dcu and K^+^ transport system.

### Specific growth rate and H_2_ production in E. coli wild type and dcu mutants at pH 7.5 and pH 5.5

For revealing the role of some proteins in bacterial cell physiology during fermentation it is important to determine one of the key physiological parameters of bacteria; the specific growth rate (µ).

**Figure 3:**
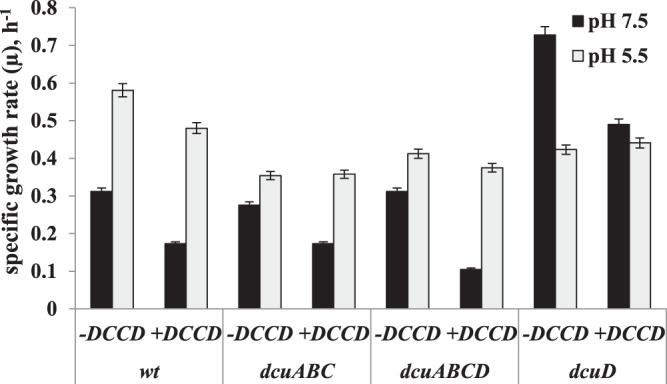
Specific growth rate (µ) of *E. coli* wild type and *dcu* mutants at pH 7.5 and pH 5.5. A parallel experiment with 0.2 mM DCCD has been performed. For strains see Table 1, for others see legends to Fig. 1.

During glycerol fermentation *E. coli* wild type cells µ was higher 1.86 fold at pH 5.5 than at pH 7.5 and yielded µ of 0.58 h^−1^. But interestingly DCCD inhibited growth at pH 7.5 but not at pH 5.5 (Fig. 3). The data are in good conformity with DCCD inhibited ATPase activity and H^+^ efflux at pH 5.5 where no inhibition was determined in wild type cells (see Figs 1B and 2). At pH 7.5 in all mutants µ was the same, as in wild type except *dcuD* one. In *dcuD* mutant µ was higher 2.3 fold, compared to wild type, whereas at pH 5.5 no such differences were found (see Fig. 3). Janausch and Unden^5^ stated that deletion of *dcuD* gene did not affect cell growth in any conditions tested, but they used glycerol plus fumarate and the metabolism goes to fumarate respiration but not to glycerol fermentation and that is why we suggest in this work that DcuD protein is presumably active under glycerol fermentative conditions. In general, it must be mentioned that mainly Dcu carriers were investigated for aerobic growth on succinate^1,2^ but fermentative conditions were not deeply studied in anaerobic conditions due to it no evidence for gene expression and thus activity of DcuD protein have been detected. Note, at pH 5.5 in all mutants µ was 1.5 fold lower than in wild type suggesting the role of Dcu carriers in cell growth.

**Figure 4:**
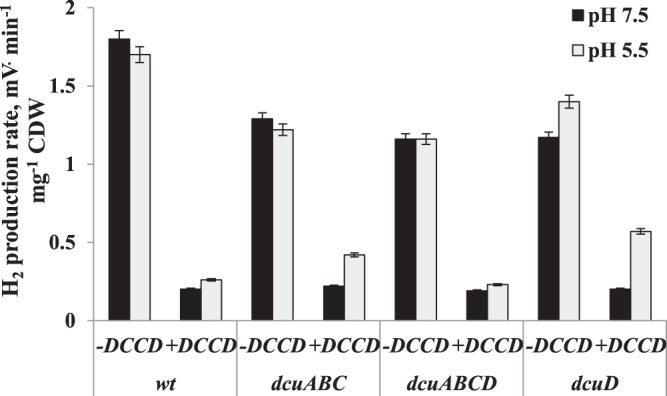
H_2_ production in *E. coli* wild type cells at pH 7.5 and pH 5.5. Cells were harvested and assayed with same concentration of glycerol as used in growth medium. The DCCD (0.2 mM) was added into the assay medium when indicated. For others see Methods section and legends to Fig. 1.

As it was stated that deletions in Dcu system have some effects on H^+^ flux and ATPase activity and it was established before that F_O_F_1_-ATPase has some link or relationship with Hyd enzymes, H_2_ production in *dcu* mutants was detected. When cells were grown on glycerol and in the assays glycerol was added, no differences were found in mutants compared to wild type at both pHs, except *dcuD* mutant in which H_2_ production was higher at pH 5.5 (Fig. 4). Moreover, no H_2_ generation was detected when succinate was added in the assays (data not shown). Note, that H_2_ production was inhibited by DCCD in *E coli* wild type and in all of the *dcu* mutants at both pH values. This suggests that F_O_F_1_ interacts with H_2_ producing Hyd enzymes but the role of Dcu C_4_-dicarboxylate carriers in H_2_ generation is absent.

## Methods

### Bacteria, growth conditions, membrane vesicles

The *E. coli* strains used in this study are listed in Table 1. Bacteria were grown under anaerobic conditions at 37 °C for 24 h in highly buffered peptone medium (20 g L^−1^ peptone, 15 g L^−1^ K_2_HPO_4_, 1.08 g L^−1^ KH_2_PO_4_, 10 g L^−1^ NaCl) with glycerol (10 g L^−1^) at pH 7.5 and pH 5.5. To achieve anaerobic conditions were achieved in glass bottles with plastic press-caps were used; O_2_ was removed from the medium by autoclaving, after which the bottles were closed by press-caps and residual O_2_ was rapidly removed by the inoculum. as described elsewhere^17,28–30^. The growth medium pH was measured by a pH-meter with pH-electrode (HJ1131B, Hanna Instruments, Portugal) and adjusted using of 0.1 M HCl or 0.1 N NaOH.

**Table 1:** Characteristics of *E. coli* wild type and mutant strains used.

Strains	Genotype	Source
Wild type strain	BW25113*rrnB ΔlacZ4787 HsdR514 Δ(araBAD)567 Δ(rhaBAD)568 rph-1* (oldgenotype: *lac1*^*q*^ *rrnBT14 ΔlacZ*_*WJ16*_ *hsdR514 ΔaraBAD*_*AH33*_ *ΔrhaBAD*_*LD78*_)	Keio Collection (NBRP)^9,17,41^
*dcuABC*	BW25113 *∆dcuA∆dcuC∆dcuB::kan*	This work
*dcuABCD*	BW25113 *∆dcuA∆dcuC∆dcuB∆dcuD::kan*	This work
*dcuD*	BW25113 *∆dcuD::kan*	Keio Collection (NBRP)^9,17,41^

The bacterial specific growth rate (μ), presented as lg2/ doubling time, was calculated, as described^31^.

Membrane vesicles isolated from bacteria, which were treated with lysozyme and ethylenediaminetetraacetic acid and prepared by the osmotic lysis of spheroplasts^32^, as described previously^20,25,27,28^.

### Membrane vesicles ATPase assay

ATPase activity was determined by the amount of inorganic phosphate (P_i_) liberated in the reaction of membrane vesicles with 5 mM ATP (pH 7.5 and 5.5)^20,25,27,28^ in the assay mixture (50 mM Tris–HCl buffer (pH 7.5 and 5.5) containing 1 mM MgSO_4_) at 37 °C thermostated chamber. Note, ATPase in right-side-out vesicles can be reached by ATP due to membrane peculiarities of cells grown under the above mentioned conditions^20,28^. In contrast ATPase in in-side-out vesicles was easily reachable in the preparations, as suggested^33^. The ATPase activity was expressed in nMol P_i_ (min µg protein)^−1^. P_i_ was determined spectrophotometrically (UV–VIS spectrophotometer, Cary 60, Agilent Technologies, USA), as described^20,25,27^. Membrane vesicles were incubated with 0.1 mM DCCD (ethanol solution) for 10 min prior assays; ethanol in the final concentration of 0.5% was used, as a blank; no effect on growth and ATPase activity was observed.

The DCCD-sensitive (inhibited) ATPase activity was calculated as a difference between activities in the absence and in the presence of the inhibitor (DCCD). In the assays KCl as a source for potassium was added in the concentration of 100 mM.

### Redox potential determination and hydrogen production assay

Redox potential (E_h_) in bacteria was determined using two different redox, titanium-silicate (Ti-Si) (EO-02, Gomel State Enterprise of Electrometric Equipment (GSEEE), Gomel, Belarus) and platinum (Pt) (EPB-1, GSEEE, or PT42BNC, Hanna Instruments, Portugal) glass electrodes^9,23,34,35^. The Ti-Si-electrode measures the overall E_h_, whereas the Pt-electrode is sensitive to H_2_ under anoxic conditions^23,34–36^. H_2_ production rate (V_H2_) was calculated as the difference between the initial rates of decrease in Pt- and Ti-Si-electrodes readings and expressed in mV of E_h_ per min per mg of cell dry weight (mV. min^−1^ mg^−1^ CDW). This type of electrochemical determination of H_2_ is similar to the Clark-type electrode used by Fernandez^36^ and other researchers^37^. As a control experiment, cells without any addition of carbon sources were used where H_2_ production was absent.

The H_2_ production determination was done in the assay buffer solution (150 mM Tris-phosphate, at the indicated pH, including 0.4 mM MgSO_4_, 1 mM NaCl and 1 mM KCl) upon glycerol addition. Glycerol, as a carbon source, was added for assays at similar concentrations, as used for cell cultivation.

H_2_ generation was also verified chemically^23,35,38^ and with Durham test tubes^13^.

### Measurement of H^+^ fluxes

H^+^ fluxes by whole cells were determined by registering the changes in H^+^ activities in the medium using selective pH electrodes (Hanna Instruments)^28,39^. The electrode readings were calibrated by titration of the medium with 0.01 M HCl. Ion fluxes were expressed in mmol/min per 10^9^ cells in 1 unit of volume.

### Protein determination, chemicals and data processing

Protein concentration was measured by the method of Lowry^40^ using bovine serum albumin (BSA), as a standard.

In experiments agar, peptone, glycerol, Tris (Carl Roth GmbH, Germany), ATP (Tris salt), BSA, DCCD, lysozyme (Sigma, USA) and other chemicals of analytical grade were used.

Data obtained from 3 independent assays are averaged, and standard deviations of values are calculated and they do not exceed 3% if not shown. Student criteria (*p*-value) is applied to validate the statistical differences in average data between various series of experiments, as described previously^29,30^; the difference is valid when *p* < 0.05 or less if not given.
